# Modelling dynamical 3D electron diffraction intensities. I. A scattering cluster algorithm

**DOI:** 10.1107/S2053273323010689

**Published:** 2024-01-25

**Authors:** Budhika Mendis

**Affiliations:** a Department of Physics, Durham University, South Road, Durham, DH1 3LE, United Kingdom; Helmholtz Centre for Infection Research, Germany

**Keywords:** dynamical electron diffraction, Bloch waves, structure matrix, multislice

## Abstract

Three-dimensional electron diffraction (3D-ED) is a powerful technique for crystallographic characterization of nanometre-sized crystals that are too small for X-ray diffraction. For accurate crystal structure refinement, however, it is important that the Bragg diffracted intensities are treated dynamically. Bloch wave simulations are often used in 3D-ED, but can be computationally expensive for large unit cell crystals due to the large number of diffracted beams. Proposed here is an alternative method, the ‘scattering cluster algorithm’ (SCA), that replaces the eigen-decomposition operation in Bloch waves with a simpler matrix multiplication. The underlying principle of SCA is that the intensity of a given Bragg reflection is largely determined by intensity transfer (*i.e.* ‘scattering’) from a cluster of neighbouring diffracted beams. However, the penalty for using matrix multiplication is that the sample must be divided into a series of thin slices and the diffracted beams calculated iteratively, similar to the multislice approach. Therefore, SCA is more suitable for thin specimens. The accuracy and speed of SCA are demonstrated on tri-iso­propyl silane (TIPS) pentacene and rubrene, two exemplar organic materials with large unit cells.

## Abstract 2

A new method is developed for calculating dynamical electron diffraction intensities.

## 1. Introduction

The wide range of electron diffraction tomography techniques, known collectively as either 3D electron diffraction (3D-ED) or MicroED, have demonstrated considerable success in solving complex structures of crystals that are otherwise too small for X-ray methods (Kolb *et al.*, 2007 ▸; Shi *et al.*, 2013 ▸; Gemmi *et al.*, 2019 ▸; Jones *et al.*, 2018 ▸). Care must be taken to minimize dynamical electron diffraction, for example by avoiding major crystal zone axes where scattering is strong, as well as integrating a given Bragg reflection over its rocking beam pattern. The latter can be achieved through either continuous rotation of the specimen holder (Nederlof *et al.*, 2013 ▸), or rotation of the holder in discrete steps combined with precession (Mugnaioli *et al.*, 2009 ▸) or tilting (Zhang *et al.*, 2010 ▸) of the electron beam. In the case of beam precession, it is known that the diffracted intensities approach the kinematic values assumed in most structure refinement models, although residual dynamical effects can still remain, especially for thicker samples and/or smaller precession angles (Own *et al.*, 2006 ▸; White *et al.*, 2010 ▸). Hence, the quality of structure refinement is improved if the intensities are treated dynamically (Palatinus *et al.*, 2015*a* ▸; Klar *et al.*, 2023 ▸), to the extent that detection of hydrogen atoms is possible (Palatinus *et al.*, 2017 ▸).

There are two widely used techniques for simulating high-energy dynamical electron diffraction, namely multislice (Jansen *et al.*, 1998 ▸) and Bloch wave (Palatinus *et al.*, 2015*b* ▸; Cleverley & Beanland, 2023 ▸) methods. Both are derived from the fundamental Schrödinger equation for a high-energy electron in a crystal (Kirkland, 2010 ▸), the key difference being the manner in which the simulations are implemented. In multislice the specimen is divided into a series of thin slices along the thickness direction, with transmission and propagation of the electron beam calculated for each slice until the specimen exit surface is reached (Cowley & Moodie, 1957 ▸). On the other hand, the Bloch wave method is based on Bloch’s theorem for the electron wavefunction in a periodic potential (Kittel, 2005 ▸), the characteristic equation for the Bloch wave being expressed as an eigenvalue problem (Spence & Zuo, 1992 ▸; Hirsch *et al.*, 1965 ▸). Multislice is faster than Bloch waves for thin specimens of large unit cell crystals, and can also deal with complex specimen shapes such as nanoparticles. However, it is not practical for 3D-ED electron tomography simulations, since a separate supercell must be generated at each specimen tilt, which must be periodic (the multislice algorithm relies on Fourier transforms) or else sufficiently large so that aliasing artefacts are minimized. Simulating different specimen orientations is comparatively easier with Bloch waves, and furthermore, analytical expressions can be used for crystal structure refinement (Palatinus *et al.*, 2015*b* ▸), thus speeding up the analysis.

Although the Bloch wave technique is the method of choice for 3D-ED, it is still limited by the relatively slow eigen-decomposition routine required for calculating Bloch wavefunctions. This is especially true for large unit cell crystals, which require a larger matrix size for accurate results. In an electron tomography simulation, eigen decomposition must also be repeated at each specimen rotation, as well as the different incident-beam directions if the electron beam is being precessed or tilted. For precession, as many as a few hundred incident-beam directions are required to achieve numerical convergence (Own *et al.*, 2006 ▸). Thus, the simulation time for Bloch waves can become prohibitively expensive. To overcome this, we propose here a novel ‘scattering cluster algorithm’ (SCA) that retains the benefits of both multislice (fast computing time for thin specimens) and Bloch waves (no supercell required), and is therefore ideally suited for electron tomography simulations. The underlying principle of SCA is that only intensity transfer or ‘scattering’ from neighbouring reflections around a given Bragg beam, *i.e.* the ‘scattering cluster’, can significantly contribute to the intensity of that beam. Eigen decomposition is replaced with a simpler matrix multiplication operation, but the cost of this is that the beam intensity must be calculated at successive specimen depths in sequence, similar to multislice. The scattering cluster algorithm works best for thin specimens of large unit cell crystals consisting of light elements. This includes biological macromolecules, functional organic materials and pharmaceuticals.

The paper is organized as follows. In Section 2 the theory behind the scattering cluster algorithm is presented, with details of the simulation given in Section 3. Tri-iso­propyl silane (TIPS) pentacene and rubrene, two workhorse organic electronic materials, are used to demonstrate the potential of the scattering cluster algorithm. In Section 4 the performance of SCA, *i.e.* accuracy and speed, is compared against standard multislice and Bloch wave simulation methods. Major zone axes, where dynamical scattering is strongest, are simulated as an extreme test of the robustness of the technique, although in an actual 3D-ED experiment zone axes are to be avoided if possible. Both normal beam incidence and precession electron diffraction patterns are simulated.

## 2. Scattering cluster algorithm

The Bloch wave characteristic equation is given by (Spence & Zuo, 1992 ▸)

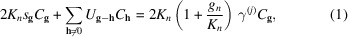

where γ^(*j*)^ and *C*_**g**_ are, respectively, the eigenvalue and Fourier coefficient of the Bloch wavefunction. *U*_**g**_ is directly proportional to the Fourier coefficient *V*_**g**_ of the crystal potential, *i.e.* *V*_**g**_ = *h* ^2^ *U*_**g**_/2*me*, where *h* is Planck’s constant and *m* and *e* are the electron mass and charge, respectively. *g*_*n*_ and *K*_*n*_ are the projections of, respectively, the reciprocal vector **g** and incident wavevector **K** along the specimen unit surface normal **n**. **K** is corrected for the mean inner potential of the crystal (Spence & Zuo, 1992 ▸), although this is usually a small change compared with the wavevector in a vacuum. The deviation parameter *s*_**g**_ is determined by the electron beam illumination,

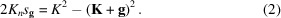




In electron diffraction tomography *g*_*n*_ and *K*_*n*_ are constantly changing with specimen tilt, but due to the short wavelength of the incident electrons and large missing wedge angle, 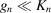 at all times for a parallel-sided specimen. As an example, for *g*_*n*_ = 1 Å^−1^ and 60° maximum tilt, *g*_*n*_/*K*_*n*_ = 0.05 for 200 kV electrons. Equation (1) can therefore be simplified into the matrix form

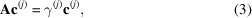

where **A** is the so-called ‘structure matrix’ and **c** ^(*j*)^ is a column vector of Bloch wave Fourier coefficients (bold font is used for vectors and matrices). The matrix elements of the former are given by

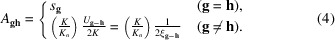

ξ_**g**_ is the extinction distance, which can be calculated from the structure factor *F*_**g**_,

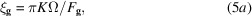




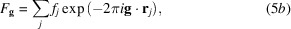

where Ω is the unit cell volume and the summation in equation (5*b* [Disp-formula fd5b]) is carried out over all atoms in the unit cell with position vector **r**_*j*_ (expressed in fractional coordinates) and atom scattering factor *f*_*j*_. Since the crystal potential is real, it follows that *U*_**g**−**h**_ = 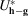 , and therefore *A*_**gh**_ = 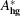 , where the asterisk denotes the complex conjugate.

The diffracted beams at depth *z* within the specimen can be expressed as

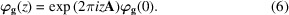


**φ**_**g**_ is a column vector of the diffracted-beam wavefunctions. The matrix exponential is most conveniently evaluated using eigenvalues γ^(*j*)^ and eigenvectors **c** ^(*j*)^ (Spence & Zuo, 1992 ▸). It is nevertheless instructive to consider the series expansion of equation (6),

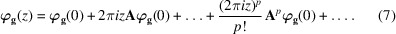

Each term in the expansion represents different orders of scattering. The following iterative equation is valid for the *n*th-order scattered Bragg reflection 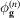 :

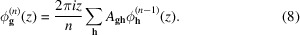




**Figure 1:**
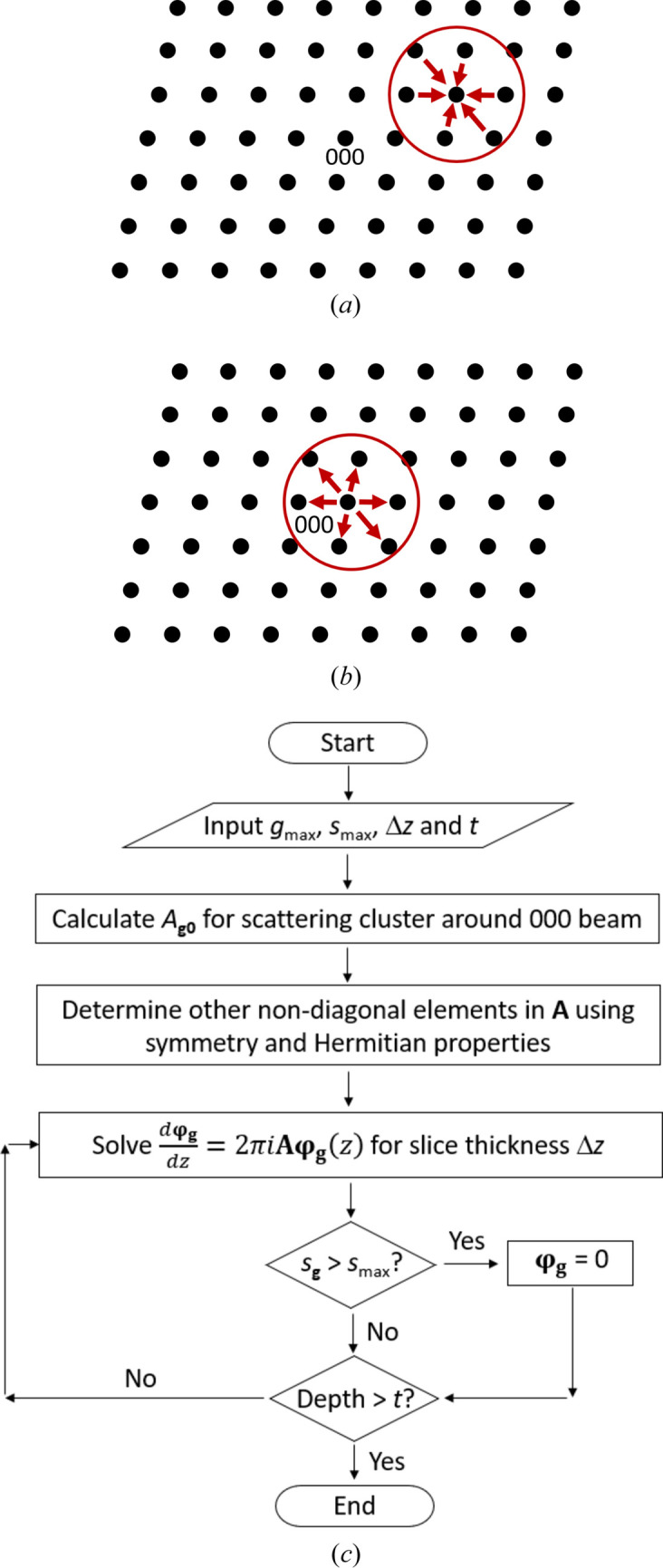
(*a*) A schematic of the principle behind the scattering cluster algorithm. The intensity of a Bragg reflection depends on scattering from neighbouring diffracted beams, *i.e.* the scattering cluster (red circle) centred around the reflection of interest. Panel (*a*) is symmetry-related to scattering from the 000 beam to Bragg reflections within an equivalent cluster centred around the reciprocal-lattice origin, as shown in (*b*). (*c*) A flowchart for the scattering cluster algorithm.

Equation (8) has a straightforward physical interpretation: it implies that 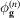 is determined by ‘cross-scattering’ of all 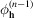 beams via the scattering potential *U*_**g**−**h**_ for **g** ≠ **h**, as well as ‘self-scattering’ of 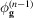 by the deviation parameter *s*_**g**_ [equation (4)]. This is illustrated schematically in Fig. 1 ▸(*a*). Generally speaking, *U*_**g**−**h**_ decreases with reciprocal distance |**g** − **h**|, albeit non-monotonically, so that scattering from only a ‘cluster’ of beams centred around the Bragg beam of interest (**g**) need be considered. The cluster will be smaller if the solid consists only of light elements, since then the magnitude of *U*_**g**−**h**_ decreases faster. The non-diagonal structure matrix terms *A*_**gh**_ depend only on the vector difference **g** − **h** [equation (4)]. Therefore, it is only necessary to calculate non-diagonal *A*_**g**0_ terms for a cluster centred around the unscattered beam [Fig. 1 ▸(*b*)]. *A*_**g**0_ terms outside the cluster are set to zero. The corresponding non-diagonal terms for the cluster centred around a Bragg beam readily follow by symmetry. The Hermitian property of the structure matrix, *i.e.* *A*_**gh**_ = 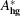 , further reduces the number of ‘unique’ non-diagonal terms that must be calculated. As an example, for **g** = 111 and **h** = 112 the vector difference **g** − **h** = 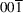 , so that *A*_**gh**_ = *A*_111, 112_ = 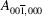 = 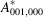 .

Although the scattering cluster concept does not alter the size of the structure matrix **A**, it does simplify computation of the important non-diagonal terms, which can be computationally expensive for large unit cells with a large number of diffracted beams. However, this is a one-off computing cost, since, apart from a trivial geometric factor in the form of *K*_*n*_ [equation (4)], the non-diagonal terms do not need re-calculating during the course of an electron diffraction tomography simulation. The computationally intensive part is finding the eigen decomposition of the structure matrix [equation (3)], which can then readily be used to calculate equation (6). The series expansion in equation (7) does not require solving equation (3), but we have found that this series converges extremely slowly, too slowly to be of any practical use. A more appropriate method is to express equation (6) in differential form (Hirsch *et al.*, 1965 ▸):

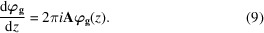




Note that equation (9) represents single scattering from the scattering cluster, unlike equations (6) and (7) which include multiple scattering, so that potentially all beams can contribute to the intensity of a given Bragg reflection. Furthermore, equation (9) requires only a matrix multiplication, which is considerably faster than eigen decomposition. The disadvantage is that we obtain only the gradient in **φ**_**g**_. To obtain **φ**_**g**_ at the specimen exit surface, equation (9) must be numerically integrated throughout the specimen depth, which is a similar procedure to multislice. For thin specimens and large unit cell crystals, however, multislice can be computationally more efficient than a standard Bloch wave calculation (Kirkland, 2010 ▸). Therefore, the scattering cluster algorithm (SCA), which is based on equation (9), could also be similarly efficient.

Implementation of the scattering cluster algorithm requires three input parameters, namely the cluster radius size *g*_max_ in reciprocal space, the real-space slice thickness Δ*z* and the maximum deviation parameter *s*_max_. *g*_max_ is determined by how fast the scattering potential decays away from the reciprocal-space origin. Δ*z* must be sufficiently small to ensure that equation (9) gives convergent results when integrated over the specimen depth. In Section 4 it will be shown that Bragg beams with larger deviation parameters require a smaller Δ*z* for convergence. It is therefore appropriate to place an upper limit *s*_max_ on the deviation parameter magnitude. Reflections with larger *s*_**g**_ values are assigned an intensity of zero and are not included in the calculation. So long as *s*_max_ is suitably chosen, there will be a negligible effect on the final result, since reflections far away from the Bragg condition are inherently weak. A flowchart of the scattering cluster algorithm is shown in Fig. 1 ▸(*c*).

Table 1 ▸ gives a quantitative comparison of the computational costs for Bloch wave, multislice and SCA in a continuous-rotation 3D-ED simulation. In Bloch waves, the computational cost for eigen decomposition of the *N*_beam_ × *N*_beam_ square matrix **A** is 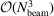 , where *N*_beam_ is the number of beams (Kirkland, 2010 ▸). The time taken to calculate the non-diagonal elements of the structure matrix is not considered, since it is performed only once at the start of the diffraction tomography simulation and can thereafter be used repeatedly with slight modification. Multislice involves fast Fourier transforms (FFTs) and matrix multiplications, which have complexity 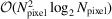 and 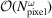 , respectively (Kirkland, 2010 ▸). The multislice diffraction patterns are assumed to be *N*_pixel_ × *N*_pixel_ square, where *N*_pixel_ must be a power of two for FFT operations. The exponent ω in square matrix multiplication is between 2 and 3. Standard square matrix multiplication requires 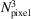 multiplication steps. However, it is possible to use fewer steps (Strassen, 1969 ▸) and the current best algorithms have ω ≃ 2.4. Furthermore, FFT and matrix multiplications are performed for each slice within the specimen, so that the computational cost increases linearly with the total number of slices *Z*_MS_. The cost of calculating projected potentials, *i.e.* 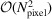 , must also be included, since this must be performed at each specimen tilt.

For the scattering cluster algorithm, equation (9) multiplies the square matrix **A** with the column vector **φ**_**g**_, which requires 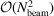 multiplication steps. This is repeated for the total number of slices *Z*_SCA_ within the specimen. Note that the number of slices for the scattering cluster algorithm need not be the same as for multislice, since the two converge at different rates. The comparison of computational costs in Table 1 ▸ suggests that the scattering cluster algorithm is faster than Bloch waves, provided the number of slices is less than the number of beams, *i.e.* *Z*_SCA_ < *N*_beam_.

## 3. Simulation method

**Figure 2:**
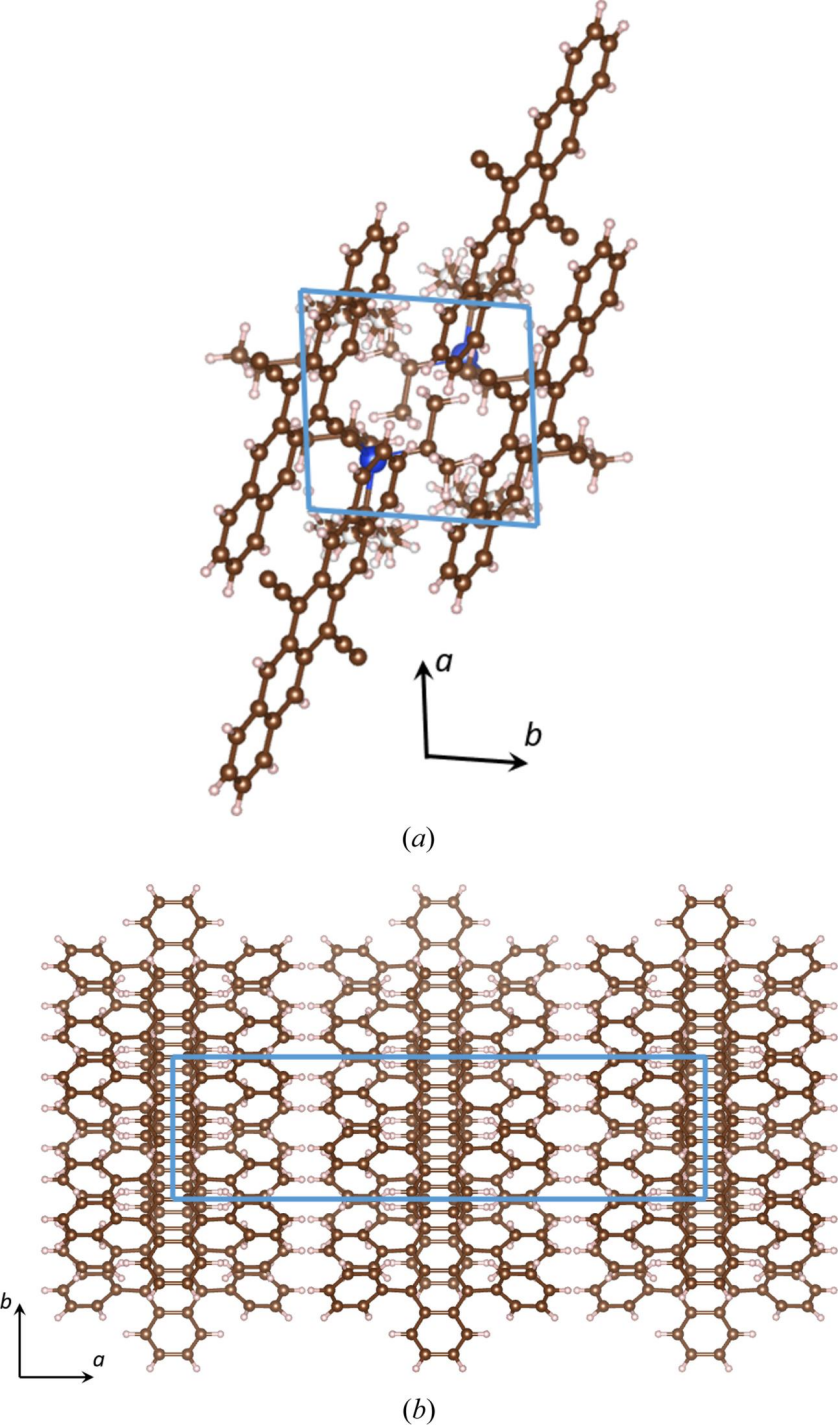
[001] projections of (*a*) TIPS pentacene and (*b*) rubrene crystal structures. The blue parallelogram is an outline of the unit cell. *a* and *b* crystallographic axes are also indicated. Carbon, hydrogen and silicon atoms are shown as brown, pink and blue spheres, respectively.

Normal beam incidence and precession electron diffraction patterns at 200 kV were simulated for [001]-oriented TIPS pentacene and rubrene using Bloch wave, multislice and SCA methods. Kirkland’s (2010 ▸) atom scattering factors were used throughout. TIPS pentacene has a triclinic crystal structure with lattice parameters *a* = 7.565 Å, *b* = 7.750 Å, *c* = 16.835 Å, α = 89.15°, β = 78.42° and γ = 83.63° (Anthony *et al.*, 2001 ▸). Rubrene has an orthorhombic crystal structure with lattice parameters *a* = 26.789 Å, *b* = 7.170 Å and *c* = 14.211 Å (Jurchescu *et al.*, 2006 ▸). Fig. 2 ▸ shows projections of the TIPS pentacene and rubrene crystal structures along the [001] axis. Both materials consist of light elements carbon and hydrogen, while TIPS pentacene also has a few silicon atoms [indicated in blue in Fig. 2 ▸(*a*)]. The atom coordinates within the unit cell were extracted from the corresponding CIF files using the *VESTA* software (Momma & Izumi, 2008 ▸) and used for calculating the non-diagonal structure matrix terms [equations (4), (5*a* [Disp-formula fd5a]) and (5*b* [Disp-formula fd5b])], as well as for constructing the multislice supercells. The simulations in this work model only elastic scattering, and do not take into account phonon (Loane *et al.*, 1991 ▸) or plasmon scattering (Mendis, 2019 ▸). The role of inelastic scattering on Bragg beam intensities is considered in the accompanying paper (Mendis, 2024 ▸).

For Bloch wave and SCA methods, the ‘projection approximation’ (Spence & Zuo, 1992 ▸) is assumed. This states that high-energy electron diffraction is largely governed by zero-order Laue zone (ZOLZ) Fourier coefficients of the crystal potential. However, since both TIPS pentacene and rubrene have large *c*-axis lattice parameters, the projection approximation may not be valid for the [001] diffraction pattern. To test this, Bloch wave calculations were performed with only ZOLZ plane Fourier coefficients in TIPS pentacene, and compared with simulations that included both ZOLZ and higher-order Laue zone (HOLZ) reflections. The results were similar, both for normal-incidence and precessed beams. All Bloch wave and SCA results reported in this paper therefore assumed the projection approximation. For TIPS pentacene, the Bragg intensities for normal beam incidence had converged for a total of 441 ZOLZ reflections, which span a ±10**g**_100_ × ±10**g**_010_ rectangular grid, where **g**_100_ and **g**_010_ are reciprocal vectors for the (100) and (010) crystal planes, respectively. Precession electron diffraction patterns, however, contain many more reflections (Own *et al.*, 2006 ▸) and therefore the number of ZOLZ reflections was increased to 1681 (*i.e.* a ±20**g**_100_ × ±20**g**_010_ grid), which is large enough for the 2° beam precession angle assumed in the simulations. The precession cone was divided into a total of 500 incident wavevectors with a uniform step size in azimuthal angle. The Bragg beam intensities for the different incident wavevectors were incoherently summed and averaged. For rubrene, the number of ZOLZ reflections was 1105 (±32**g**_100_ × ±8**g**_010_) and 4257 (±64**g**_100_ × ±16**g**_010_), respectively, for normal-incidence and 2° precession angle simulations. The higher sampling along **g**_100_ was due to the larger *a*-axis lattice parameter for the rubrene unit cell.

For multislice, the TIPS pentacene supercell had a (square) side length of 54.25 Å (= 7*b*) in the plane of the specimen and a slice thickness of 1.05 Å (= *c*/16). The lateral dimensions must be sufficiently large to minimize aliasing artefacts from non-periodic boundary conditions, which is especially important for precession diffraction, since the incident beam is highly tilted. For a 1024 × 1024 pixel diffraction pattern, the bandwidth-limited maximum scattering angle is 9°, which is significantly larger than the 2° precession angle. For higher multislice accuracy the slice thickness must be as small as possible, although it must also be sufficiently thick to encompass the full projected potential of atoms within the slice. For the organic materials in this study, a slice thickness of ∼1 Å was chosen as a suitable compromise. Simulations performed with thicker slices of 2.10 Å (= *c*/8) produced similar results, indicating that the results had converged with respect to slice thickness. Artefacts in the HOLZ beam intensities can appear if the crystal periodicity in the specimen thickness direction is not an integer multiple of the slice thickness (Kilaas *et al.*, 1987 ▸), although this is not important for the present study. For rubrene the supercell had a (square) side length of 136.23 Å (= 19*b*) in the plane of the specimen and a slice thickness of 1.02 Å (= *c*/14). Larger supercell dimensions are required to prevent overlapping of the 100 systematic reflections, while maintaining periodic boundary conditions as much as possible (recall that the *a* and *b* lattice parameters for rubrene are very different). Consequently, the bandwidth-limited maximum scattering angle is reduced to 3.6° for 1024 × 1024 pixel sampling, although this is still larger than the 2° precession angle.

Multislice simulated diffraction intensities for TIPS pentacene were extracted by placing a 7×7 pixel mask around select Bragg reflections. For rubrene the mask size was reduced to 3×3 pixels to avoid overlap with neighbouring reflections along the 100 systematic row. To simulate the diffraction pattern, the multislice electron wavefunction was multiplied by a Hanning window before calculating its power spectrum. This minimizes aliasing artefacts due to non-periodicity in the supercell and/or incident beam. For precession the cone of incident wavevectors is discretely sampled in uniform azimuthal angle steps. A total of 500 incident wavevectors were simulated. Individual diffraction patterns were aligned to remove beam tilt and averaged to give the overall precession electron diffraction pattern. All simulations were run in *MATLAB* and CPU times for a standard 8 GB RAM desktop PC were recorded using the tic toc command.

## 4. Results and discussion

**Figure 3:**
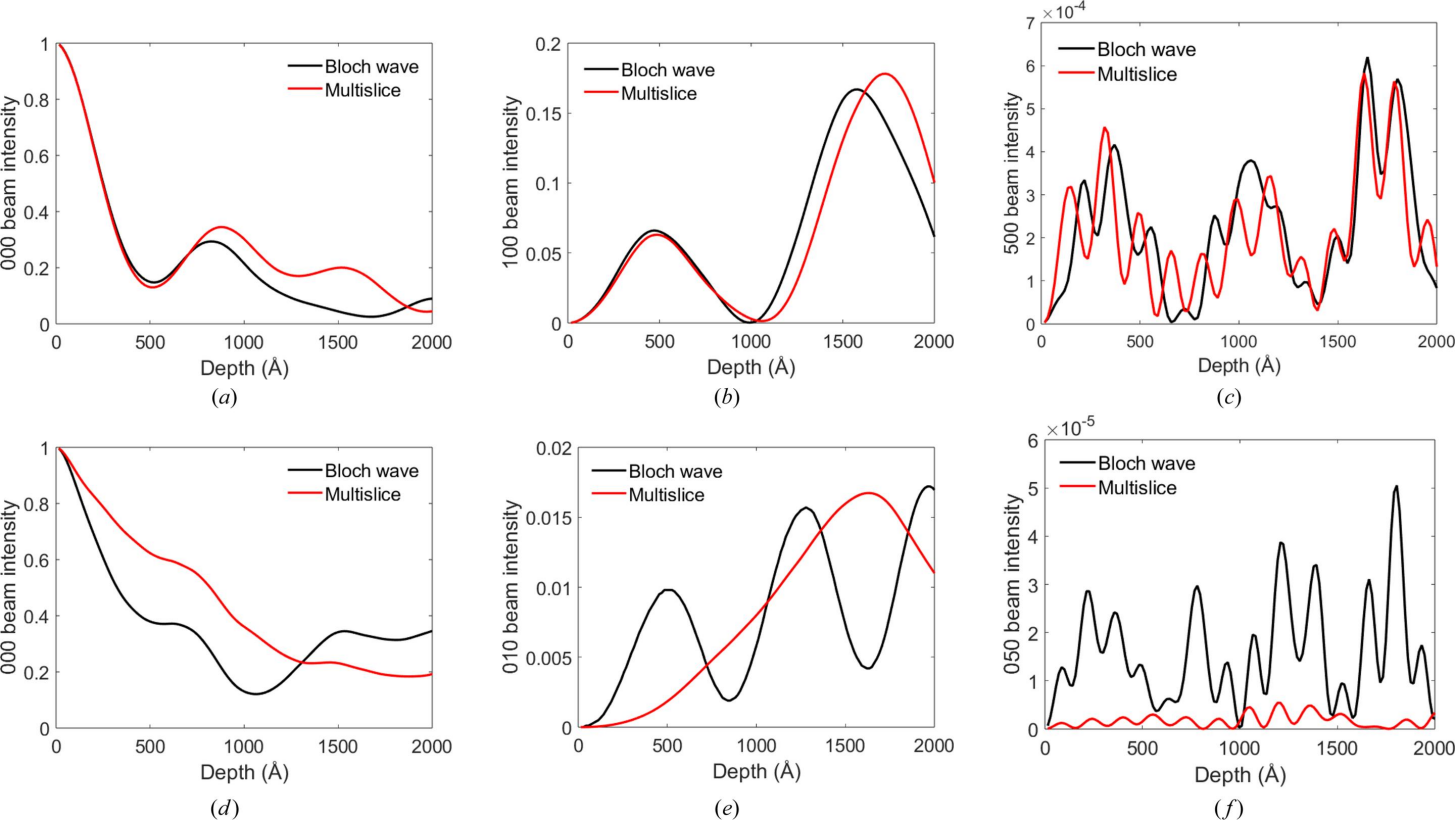
Bloch wave and multislice simulated intensity pendellösung data for normal beam incidence in [001]-TIPS pentacence and rubrene. For TIPS pentacene the intensities are plotted for (*a*) the 000, (*b*) the 100 and (*c*) the 500 reflections, while for rubrene the intensities correspond to (*d*) the 000, (*e*) the 010 and (*f*) the 050 beams. The total electron intensity is normalized to unity.

We first compare results for the traditional Bloch wave and multislice simulation methods. Fig. 3 ▸ shows intensity pendellösung data for select Bragg beams in [001]-oriented TIPS pentacene and rubrene at normal electron beam incidence. The Bragg beams chosen are the unscattered 000 beam, a low-index Bragg beam at ∼0.1 Å^−1^ reciprocal distance (*i.e.* 100 and 010 in TIPS pentacene and rubrene, respectively) and a high-index Bragg beam at ∼0.7 Å^−1^ (*i.e.* 500 and 050 in TIPS pentacene and rubrene, respectively). This choice of Bragg beams covers a large dynamic range and provides a structural resolution better than 1.5 Å. For TIPS pentacene there is satisfactory agreement between the Bloch wave and multislice simulated pendellösung data [Figs. 3 ▸(*a*)–3 ▸(*c*)]. However, for rubrene all reflections show substantial discrepancies [Figs. 3 ▸(*d*)–3 ▸(*f*)]. As indicated in Section 3, the convergence of the Bloch wave results was tested with respect to the number of reflections (ZOLZ and HOLZ), while the multislice results converged with respect to the supercell lateral dimensions, number of pixels and slice thickness. Therefore, any differences in the pendellösung data must be due to systematic errors between the two methods, rather than numerical convergence. Systematic errors in conventional multislice, and strategies to mitigate them, have been discussed in detail by Chen & Van Dyck (1997 ▸) and Chen *et al.* (1997 ▸). Improved multislice algorithms have not been investigated here, since they are not the main topic of interest in this work. Instead, it is assumed that the Bloch wave results are accurate and can therefore be used as a reference for testing the validity of the scattering cluster algorithm.

**Figure 4:**
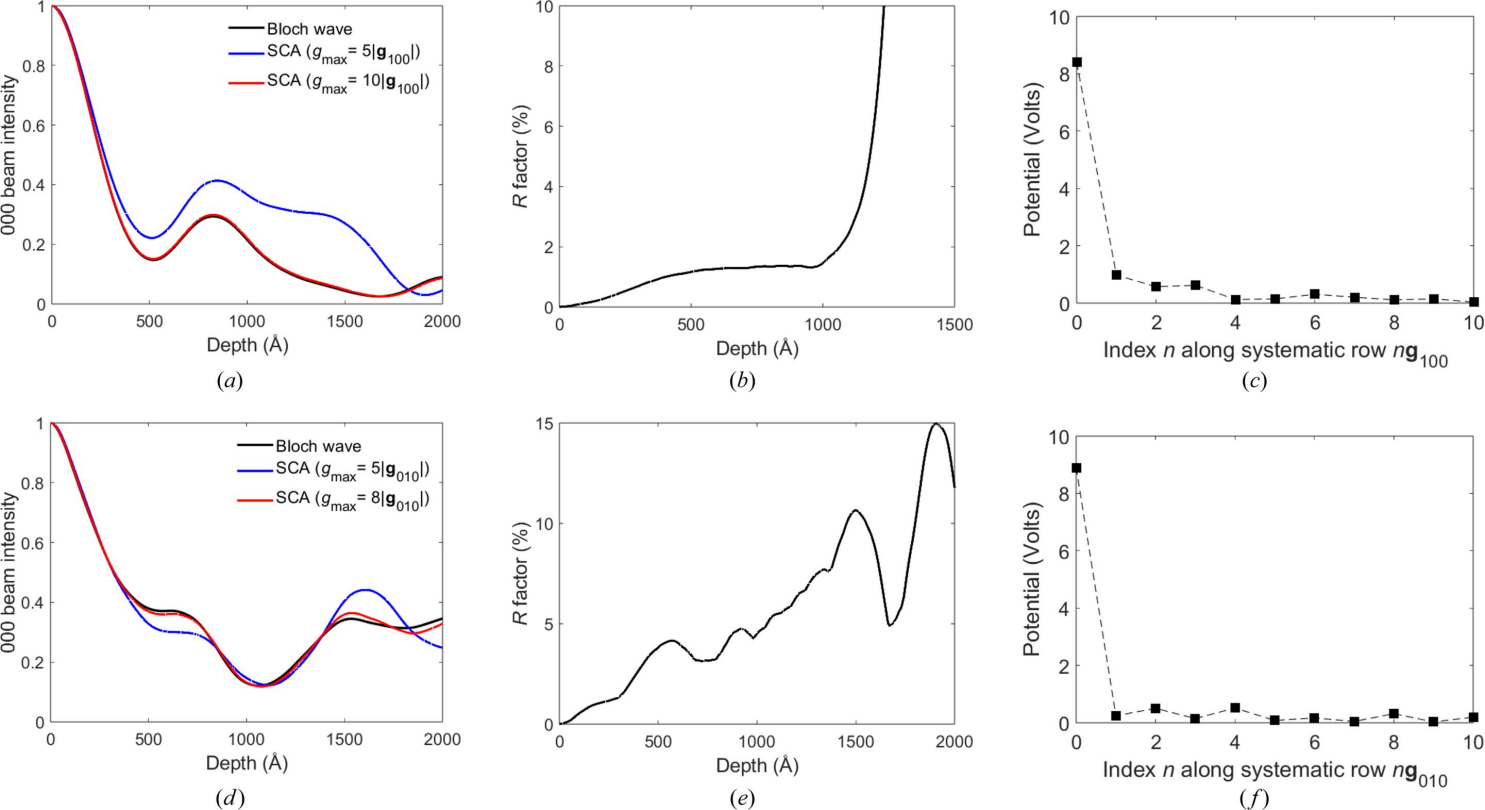
(*a*) A comparison of SCA-calculated 000 beam intensity pendellösung data in [001]-TIPS pentacene with Bloch wave results (normal beam incidence). SCA results for two different *g*_max_ values are presented, *i.e.* 5|**g**_100_| and 10|**g**_100_|. (*b*) *R* factor plotted as a function of specimen depth for the 10|**g**_100_| cluster-size simulation. The *R* factor increases rapidly beyond ∼1000 Å (for visual clarity, the vertical scale is truncated to 10%). (*c*) The Fourier component of the TIPS pentacene crystal potential along the 100 systematic row. (*d*) A comparison of SCA-calculated 000 beam intensity pendellösung fringes in [001]-rubrene with Bloch wave results (normal beam incidence). SCA results for two different *g*_max_ values are presented, *i.e.* 5|**g**_010_| and 8|**g**_010_|. (*e*) *R* factor plotted as a function of specimen depth for the 8|**g**_010_| cluster-size simulation. (*f*) The Fourier component of the rubrene crystal potential along the 010 systematic row.

In Fig. 4 ▸(*a*) the convergence of the scattering cluster algorithm is compared against the Bloch wave calculation for the 000 beam pendellösung data in TIPS pentacene. There are three input parameters, *g*_max_, Δ*z* and *s*_max_, that can be varied in an SCA simulation. For simplicity, no limit was initially set on *s*_max_, *i.e.* the intensities of all Bragg reflections were calculated. A Δ*z* value of 0.2 Å produced converged results for TIPS pentacene, which is considerably smaller than the ∼1–2 Å slice thickness typically used for multislice. Larger values of Δ*z* resulted in non-physical results, *e.g.* beam intensities greater than unity, the breakdown first being observed at larger specimen depths (see the supporting information). *g*_max_ determines the number of neighbouring reflections contributing to the intensity of a given Bragg beam [Fig. 1 ▸(*a*)]. Clearly, larger values of *g*_max_ would give more accurate results. This is observed in Fig. 4 ▸(*a*), which shows SCA results for *g*_max_ values of 5|**g**_100_| (0.7 Å^−1^) and 10|**g**_100_| (1.4 Å^−1^). The numbers of reflections within a scattering cluster are 84 and 334, respectively. SCA convergence for other select Bragg beams shows a similar trend (see the supporting information). The *R* factor is used to quantify overall convergence. It is defined by *R* = 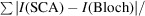 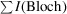 , where the summation is over all Bragg beam intensities (*I*) in the SCA and Bloch wave simulations. The dynamic range of the diffracted intensities can vary over ten orders of magnitude, and inclusion of the weakest beams in the *R* factor can sometimes lead to unusually high values. Such weak reflections are unlikely to be selected for structure refinement in a real experiment. Fig. 4 ▸(*b*) plots the *R* factor as a function of specimen depth for the larger 10|**g**_100_| cluster radius simulation. The *R* factor is below 2% for specimens thinner than 1000 Å but rapidly increases thereafter. The convergence for specimens thicker than 1000 Å can be improved by using a smaller slice thickness Δ*z* (see the supporting information).

The role of *g*_max_ and Δ*z* on numerical convergence can be understood through equation (9). In particular, *g*_max_ must be sufficiently large so that the non-diagonal elements of the structure matrix **A**, or equivalently the Fourier component of the crystal potential [equation (4)], have decreased to a negligible value. Fig. 4 ▸(*c*) shows the Fourier component of the TIPS pentacene crystal potential along the 100 systematic row. The potential decreases sharply within the first few reflections, although there is a long tail of much weaker potential. Therefore, a small cluster radius (*e.g.* 5|**g**_100_|) is sufficient to give converged results, provided the specimen is thin. At larger specimen thicknesses, however, the outer reflections at the cluster perimeter and beyond will accumulate greater intensity, and scattering from these beams cannot be ignored despite the smaller crystal potentials. Therefore, a larger cluster radius is required for convergence [Fig. 4 ▸(*a*)]. Specimens consisting of light elements have smaller crystal potentials and therefore smaller *g*_max_ values as well. Furthermore, structure matrix elements *A*_**gh**_ with large amplitude produce large changes in **φ**_**g**_ [equation (9)], so that the slice thickness Δ*z* must be made sufficiently small to ensure numerical convergence. For our simulations the diagonal elements of **A** have the largest amplitudes. The diagonal terms are proportional to the deviation parameter [equation (4)] and hence decrease with increasing electron-beam voltage and unit cell size. SCA is therefore suitable for organic materials, due to their weaker crystal potentials and relatively large unit cells.

Fig. 4 ▸(*d*) shows the 000 beam pendellösung convergence in rubrene for SCA cluster sizes of 5|**g**_010_| (0.7 Å^−1^) and 8|**g**_010_| (1.1 Å^−1^), which have 286 and 73 reflections, respectively. As expected, the results for the larger cluster are in better agreement with Bloch waves (see the supporting information for convergence of other select Bragg beams). The *R* factor [Fig. 4 ▸(*e*)] for this cluster is smaller than 5% for specimens thinner than 1000 Å, and remains within 15% for thicknesses up to 2000 Å. The overall convergence for thick specimens is therefore better than for TIPS pentacene [Fig. 4 ▸(*b*)], probably due to the smaller deviation parameters for the larger unit cell rubrene crystal. The Fourier component of the rubrene crystal potential decreases rapidly along the 010 systematic row [Fig. 4 ▸(*f*)] and is consistent with the observed SCA convergence for an 8|**g**_010_| cluster size.

**Figure 5:**
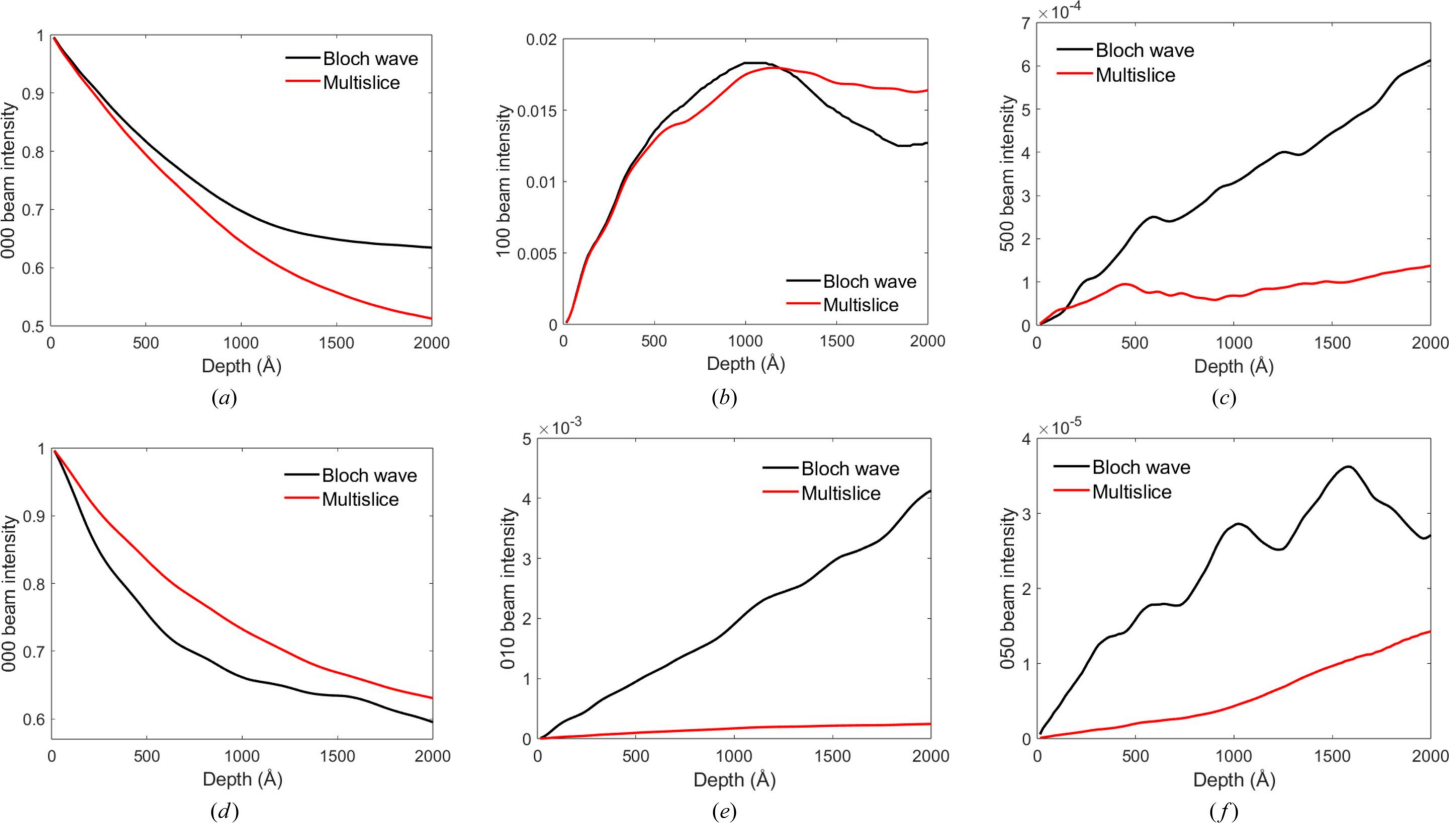
Bloch wave and multislice simulated precession intensity pendellösung data for [001]-TIPS pentacence and rubrene. The electron-beam precession angle is 2°. For TIPS pentacene, intensities are plotted for (*a*) the 000, (*b*) the 100 and (*c*) the 500 reflections, while for rubrene the intensities correspond to (*d*) the 000, (*e*) the 010 and (*f*) the 050 beams. The total electron intensity is normalized to unity.

Having established the accuracy of the SCA, we now focus on its computational efficiency. To this end, we look to precession electron diffraction, which is widely used in electron diffraction tomography (Mugnaioli *et al.*, 2009 ▸) but computationally demanding to simulate, due to the fine sampling of the beam azimuthal angle that is required to achieve convergence (Own *et al.*, 2006 ▸; White *et al.*, 2010 ▸). Improving the speed of precession electron diffraction simulations is therefore highly desirable for crystal structure refinement. Fig. 5 ▸ shows precession intensity pendellösung data for [001]-oriented TIPS pentacene and rubrene, calculated using Bloch wave and multislice methods. Intensities are plotted for the 000 unscattered beam, as well as example low- and high-index Bragg reflections (*i.e.* 100/500 and 010/050 for TIPS pentacene and rubrene, respectively). Compared with normal beam incidence (Fig. 3 ▸), the precession intensity contains fewer oscillations with respect to specimen depth, which suggests that the 2° beam precession angle has suppressed dynamical diffraction to some degree. This is especially true for small specimen depths (≲ 500 Å), where the precession intensity pendellösung data show approximately linear behaviour. Once again there are systematic differences between the Bloch wave and multislice results, which are tentatively assigned to the limited accuracy of the latter.

Next, we consider precession simulations using the SCA. *g*_max_ is set to the values established previously for normal beam incidence, *i.e.* 10|**g**_100_| for TIPS pentacene [Fig. 4 ▸(*a*)] and 8|**g**_010_| for rubrene [Fig. 4 ▸(*d*)]. During beam precession the Bragg reflections will sweep across large sections of the rocking beam pattern (Palatinus *et al.*, 2019 ▸). Therefore, Δ*z* must be made exceedingly small to achieve convergence for the very large deviation parameters encountered during precession. This makes the simulation impractical, even for reasonably thin specimens. The *s*_max_ parameter overcomes this limitation by calculating the intensities of only those reflections which satisfy |*s*_**g**_| ≤ *s*_max_ for a given incident wavevector. We have found *s*_max_ = (λ/2)(*g*_max_/2)^2^ to be a suitable value, where λ is the electron wavelength. This value corresponds to the deviation parameter magnitude at half the cluster size (*g*_max_/2) for normal beam incidence. At 200 kV *s*_max_ has values of 5.8 × 10^−3^ Å^−1^ and 3.9 × 10^−3^ Å^−1^ for TIPS pentacene and rubrene, respectively. As a guide, in two-beam dynamical diffraction, the rocking beam pattern has its first minimum at a specimen thickness *t* = 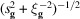 (Hirsch *et al.*, 1965 ▸). The extinction distance ξ_**g**_ is relatively large for organic materials consisting of light elements, and can therefore be ignored in the expression for *t*. For the *s*_max_ values in the present study, the simple two-beam model predicts a specimen thickness *t* of 172 Å for TIPS pentacene and 256 Å for rubrene. Specimens thinner than these values would require a larger *s*_max_ for accurate simulation.

**Figure 6:**
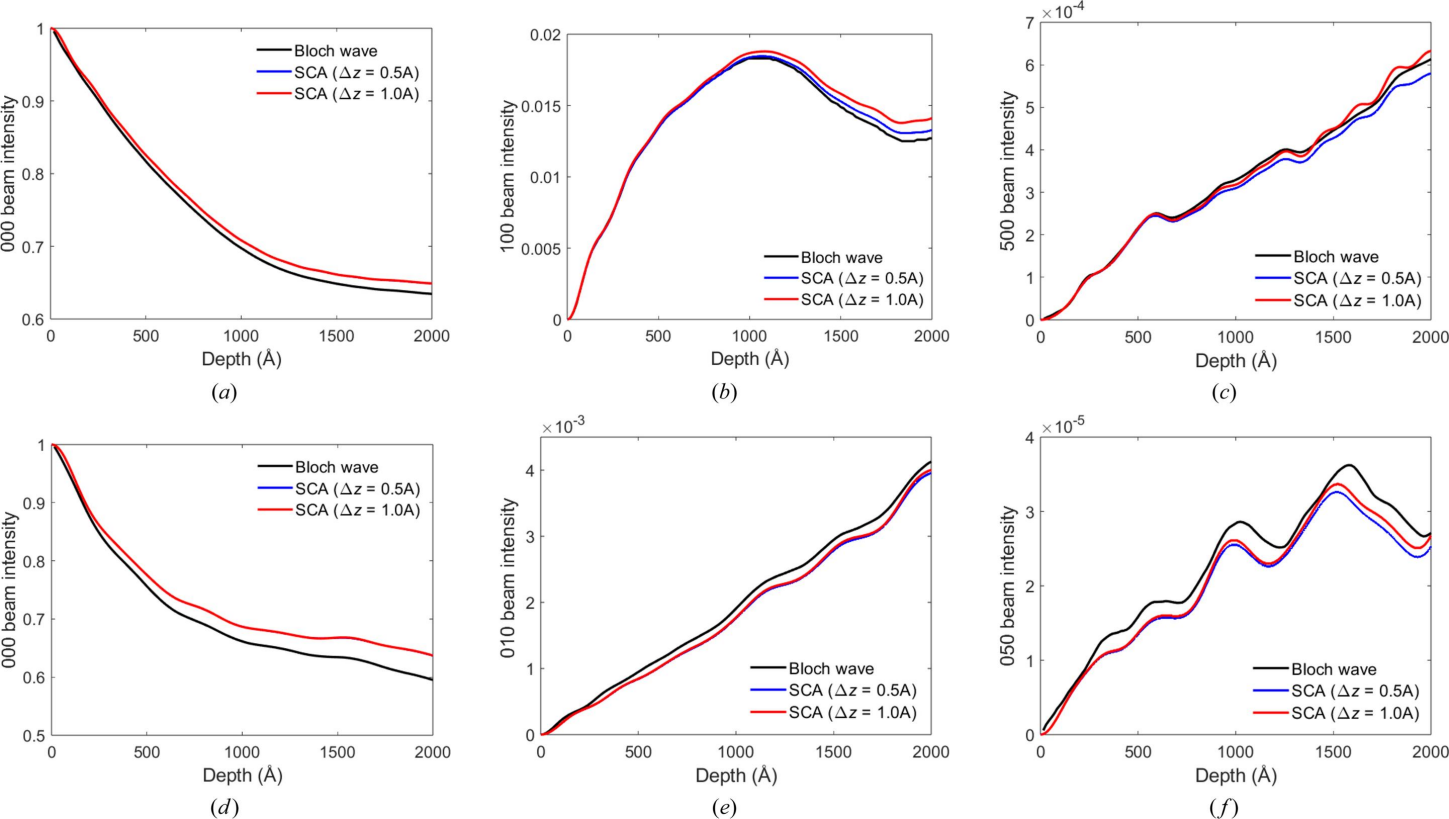
A comparison of SCA-simulated precession intensity pendellösung data for [001]-TIPS pentacene and rubrene with Bloch wave results. The electron-beam precession angle is 2°. *s*_max_ values of 5.8 × 10^−3^ Å^−1^ and 3.9 × 10^−3^ Å^−1^ were used for SCA simulations of TIPS pentacene and rubrene, respectively. The SCA slice thickness Δ*z* was either 0.5 Å or 1.0 Å. For TIPS pentacene, precession intensities are plotted for (*a*) the 000, (*b*) the 100 and (*c*) the 500 reflections, while for rubrene the intensities correspond to (*d*) the 000, (*e*) the 010 and (*f*) the 050 beams. The SCA pendellösung data for the two Δ*z* values directly overlap in panels (*a*) and (*d*). The total electron intensity is normalized to unity.

The introduction of *s*_max_ enables thicker slices Δ*z* to be used for calculating precession beam intensities. Fig. 6 ▸ compares the convergence of the SCA with Bloch wave precession intensity pendellösung data for [001]-TIPS pentacene and rubrene. A maximum deviation parameter was imposed on the SCA simulation, and results for 0.5 Å and 1.0 Å slice thicknesses are plotted. The two Δ*z* values give similar results for specimen depths below ∼1000 Å in TIPS pentacene [Figs. 6 ▸(*a*)–6 ▸(*c*)], while for rubrene the agreement extends to larger depths [Figs. 6 ▸(*d*)–6 ▸(*e*)]. Thus we do not observe the breakdown in accuracy at small depths due to a finite *s*_max_ predicted by two-beam theory. This could be because a two-beam model is an oversimplification of the more complex many-beam scattering geometry valid for a flat Ewald sphere. Furthermore, at shallow specimen depths, *i.e.* depths much smaller than ξ_**g**_/2, the diffracted beam intensities are inherently weak, so that the main scattering mechanism is due to intensity transfer from the 000 beam to the diffracted beams. This suggests that it is less important to include a larger number of diffracted beams in the scattering cluster, so that *s*_max_ need not be prohibitively large. It is clear that the chosen values for *s*_max_ produce SCA results that are in good agreement with Bloch wave calculations for a large range of specimen thicknesses (Fig. 6 ▸). The larger slice thicknesses are also similar to what is used in multislice, so that simulation times are kept reasonable.

In Table 2 ▸ the computational times for precession simulations using Bloch wave, SCA and multislice methods are compared for 500 Å and 1000 Å thick TIPS pentacene and rubrene in [001] orientation. In each case, 500 incident wavevectors were simulated to ensure convergence. The other simulation parameters are as indicated in Section 3. It is possible that less-stringent parameters could have been chosen without sacrificing much accuracy, such as fewer beams in a Bloch wave calculation, a larger Δ*z* for SCA, a smaller number of pixels in multislice *etc*. Furthermore, the *MATLAB* code in this work is an interpreted language and not optimized for speed. Therefore, the absolute values in Table 2 ▸ are not important, but what is useful are the relative trends between the different values. Bloch wave simulation times are independent of specimen thickness, while for SCA and multislice the simulation time increases monotonically with thickness (Table 1 ▸). Overall, for TIPS pentacene Bloch wave is the most efficient simulation method. SCA is slower and also has a large *R* factor (Table 2 ▸), indicating poor overall accuracy. The *R* factor can be reduced by increasing *s*_max_ and/or reducing Δ*z*, although this increases the SCA simulation time even further. The simulation times for multislice are, however, considerably longer than either Bloch wave or SCA methods. This is because of the large supercell dimensions for TIPS pentacene, which must then be sampled with a large number of pixels to accommodate the extra reflections in precession electron diffraction.

For rubrene, SCA consistently outperforms both Bloch wave and multislice methods, and has a satisfactory *R* factor (Table 2 ▸). For example, SCA simulations with 1.0 Å slice thickness are faster than Bloch waves by factors of ∼4 and 2 for 500 Å and 1000 Å thick rubrene, respectively (Table 2 ▸). In an electron tomography measurement, which contains several specimen tilts, there would be a large saving in computing time. Rubrene has a much larger number of beams than TIPS pentacene (4257 versus 1681; Section 3). Therefore, for the specimen thicknesses of interest here, the matrix multiplication operation in SCA is more efficient than Bloch wave eigen decomposition (Table 1 ▸). Larger unit cell crystals contain many more Bragg beams, resulting in a further reduction in SCA computing times compared with Bloch waves. For example, the lattice parameters for a lysozyme protein crystal are *a* = *b* = 77 Å and *c* = 37 Å (Shi *et al.*, 2013 ▸), significantly larger than rubrene. However, the thicknesses of protein crystals tend to be quite large [*e.g.* ∼5000 Å in the work of Shi *et al.* (2013 ▸)], which would reduce the efficiency of SCA. For SCA to outperform Bloch waves the unit cell must be large and the specimen thickness below a critical value, the critical thickness increasing with the number of beams in the simulation.

SCA is also considerably faster at calculating the non-diagonal terms of the structure matrix. In principle, this method of calculating the structure matrix could also be applied to Bloch waves, although not all the non-diagonal terms are evaluated. Nevertheless, as is clear from the SCA results, this ‘partial’ structure matrix is still sufficiently accurate to produce converged results. Although the non-diagonal structure matrix terms need only be calculated once in electron diffraction tomography, for large unit cell crystals with many diffracted beams this task can be computationally non-trivial. As an example, for rubrene, with 290 unit cell atoms and 4257 reflections, the time taken to calculate the non-diagonal structure matrix elements is 0.5 min for SCA and 22 min for Bloch waves. The number of non-diagonal terms is 4257^2^ − 4257 = 18 117 792, compared with only 734 non-diagonal terms calculated with SCA (ignoring the Hermitian property of the structure matrix, which would reduce the number of ‘unique’ non-diagonal terms still further). The increase in SCA computational efficiency (*i.e.* 22/0.5 = 44) is, however, considerably less than the reduction in non-diagonal terms (*i.e.* 18 117 792/734 ≃ 24 684), due to the fact that pre-calculated values must be assigned to other non-diagonal terms based on symmetry. Nevertheless, the increase in speed is still significant for rubrene, and could potentially be enhanced further by optimizing the symmetry-based assignment part of the algorithm. If the specimen is also thick, Bloch waves may be more appropriate than SCA and, in such cases, combining the SCA method for structure matrix calculation with Bloch wave eigen decomposition may provide the most efficient simulation method.

## 5. Summary

A scattering cluster algorithm (SCA) is proposed as an alternative method for calculating dynamical electron diffraction densities. SCA shares some common features with traditional Bloch wave and multislice simulation methods. The underlying principle of SCA is that the intensity of any given Bragg reflection is governed by intensity transfer or ‘scattering’ from neighbouring diffracted beams, *i.e.* the scattering cluster. Using this principle, the important non-diagonal terms in the structure matrix can be rapidly calculated, resulting in significant computational savings for large unit cell crystals, where the number of diffracted beams is large. Implementation of SCA relies on matrix multiplication, which is much faster than the eigen-decomposition routine of Bloch waves. However, the cost of matrix multiplication is that the specimen must be divided into thin slices and the wavefunction within the specimen solved iteratively, similar to multislice. Therefore, SCA will only outperform Bloch waves for ‘thin’ specimens, the crossover thickness increasing monotonically with the number of diffracted beams in the simulation. For this reason, SCA is more suitable for simulating the complex organic crystals that are of interest in 3D electron diffraction, such as biological macromolecules, organic electronic mater­ials and pharmaceuticals.

The computer code for this work is available open access from the Durham University research data repository (DOI: https://doi.org/10.15128/r29g54xh72z).

## Supplementary Material

Additional derivations and figures. DOI: 10.1107/S2053273323010689/tw5006sup1.pdf

## Figures and Tables

**Table 1:** Computational cost per specimen tilt for continuous-rotation 3D-ED simulations using Bloch wave, multislice and scattering cluster algorithm methods *N*_beam_ is the number of beams in the Bloch wave and scattering cluster algorithm, while *N*_pixel_ is the number of pixels for the side length of a (square) diffraction pattern in multislice. *Z*_MS_ and *Z*_SCA_ are the total number of slices in multislice and the scattering cluster algorithm, respectively.

Algorithm	Complexity
Bloch wave	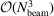
Multislice	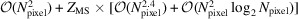
Scattering cluster	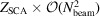

**Table 2:** Precession electron diffraction simulation times in minutes for Bloch wave, scattering cluster algorithm and multislice methods The samples are [001]-TIPS pentacene and rubrene, at two different specimen thicknesses of 500 Å and 1000 Å. Five hundred incident wavevectors were sampled uniformly over the precession cone, which had a 2° precession angle. For the scattering cluster algorithm, simulation times for two different slice thicknesses (*i.e.* 0.5 Å and 1.0 Å) are presented. The numbers within brackets are the *R* factors expressed as percentages.

		Scattering cluster algorithm		
Sample	Bloch wave	Δ*z* = 0.5 Å	Δ*z* = 1.0 Å	Multislice
TIPS pentacene (500 Å)	24.7	28.7 (1.8%)	14.6 (58.3%)	362.3
TIPS pentacene (1000 Å)	25.5	68.8 (62.5%)	31.6 (∼10^6^%)	703.4
Rubrene (500 Å)	364.4	173.7 (3.9%)	88.3 (3.9%)	368.6
Rubrene (1000 Å)	367.7	349.1 (6.0%)	179.4 (6.1%)	755.2
