# Supplementary material for: Modelling dynamical 3D electron diffraction intensities. I. A scattering cluster algorithm
Source: Acta Crystallogr A Found Adv. 2024 Jan 25;80(Pt 2):167–77. doi: 10.1107/S2053273323010689 (PMC10913674; doi:10.1107/S2053273323010689)
Supplement: Supplementary file 1 [file a-80-00167-sup1.pdf]

Acta Cryst A
FOUNDATIONS
ADVANCES

Volume 80 (2024)

Supporting information for article:

Modelling dynamical 3D electron diffraction intensities. I. A scattering cluster algorithm

Budhika Mendis

(1) Effect of slice thickness on scattering cluster algorithm convergence

Figure S1 shows the 000 beam intensity pendellösung for [001]-TIPS pentacene calculated using the scattering cluster algorithm (SCA). The electron beam is parallel to the optic axis (i.e. normal incidence) and has a total intensity of unity. Results are plotted for slice thickness (Δz) values of 0.25 Å and 0.30 Å, which are slightly larger than the 0.20 Å slice thickness used in Figure 3a (main text). Increasing the slice thickness causes a breakdown in the numerical convergence, first in the form of rapid oscillations at large thicknesses ($\gtrsim$1750 Å; Figure S1a), which on increasing Δz still further gives rise to beam intensities significantly above unity (Figure S1b).

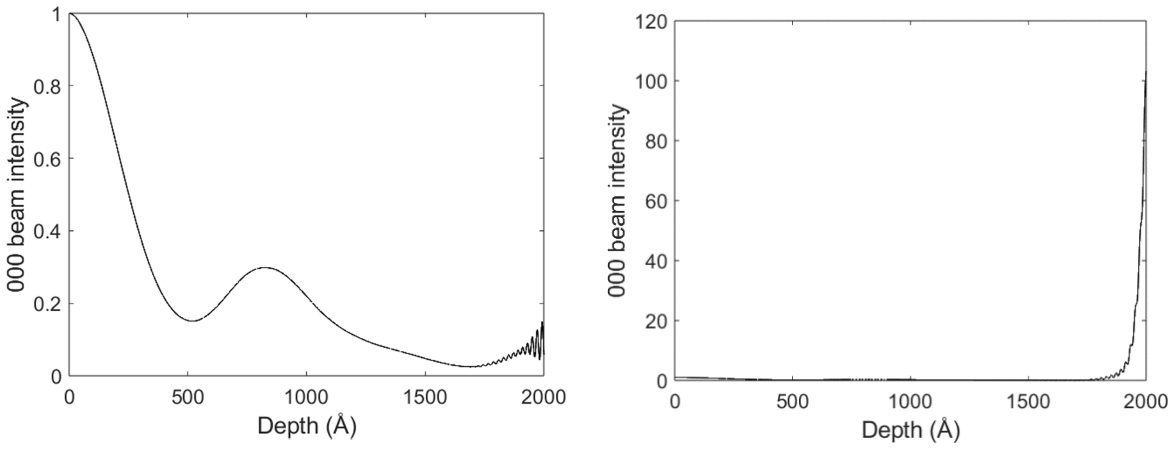

(a) (b)

Figure S1: 000 beam intensity pendellösung for [001]-TIPS pentacene calculated using the scattering cluster algorithm. The slice thickness Δz is 0.25 Å in (a) and 0.30 Å in (b).

(2) Comparison of scattering cluster algorithm and Bloch wave intensities

Figure S2 compares intensity pendellösung for [001]-TIPS pentacene and rubrene calculated using Bloch waves and SCA. The electron beam is parallel to the optic axis (i.e. normal incidence) and has a total intensity of unity. Pendellösung for example low and high index reflections, i.e. 100/010 and 500/050 for TIPS pentacene/rubrene respectively, are plotted. Furthermore, SCA results for two different $g_{\max}$ values are plotted, i.e. $5|\mathbf{g}_{100}|$ and $10|\mathbf{g}_{100}|$

for TIPS pentacene, and $5|\mathbf{g}_{010}|$ and $8|\mathbf{g}_{010}|$ for rubrene respectively. The SCA slice thickness Δz is 0.2 Å. In each case, the larger $g_{\max}$ value is in close agreement with the Bloch wave result.

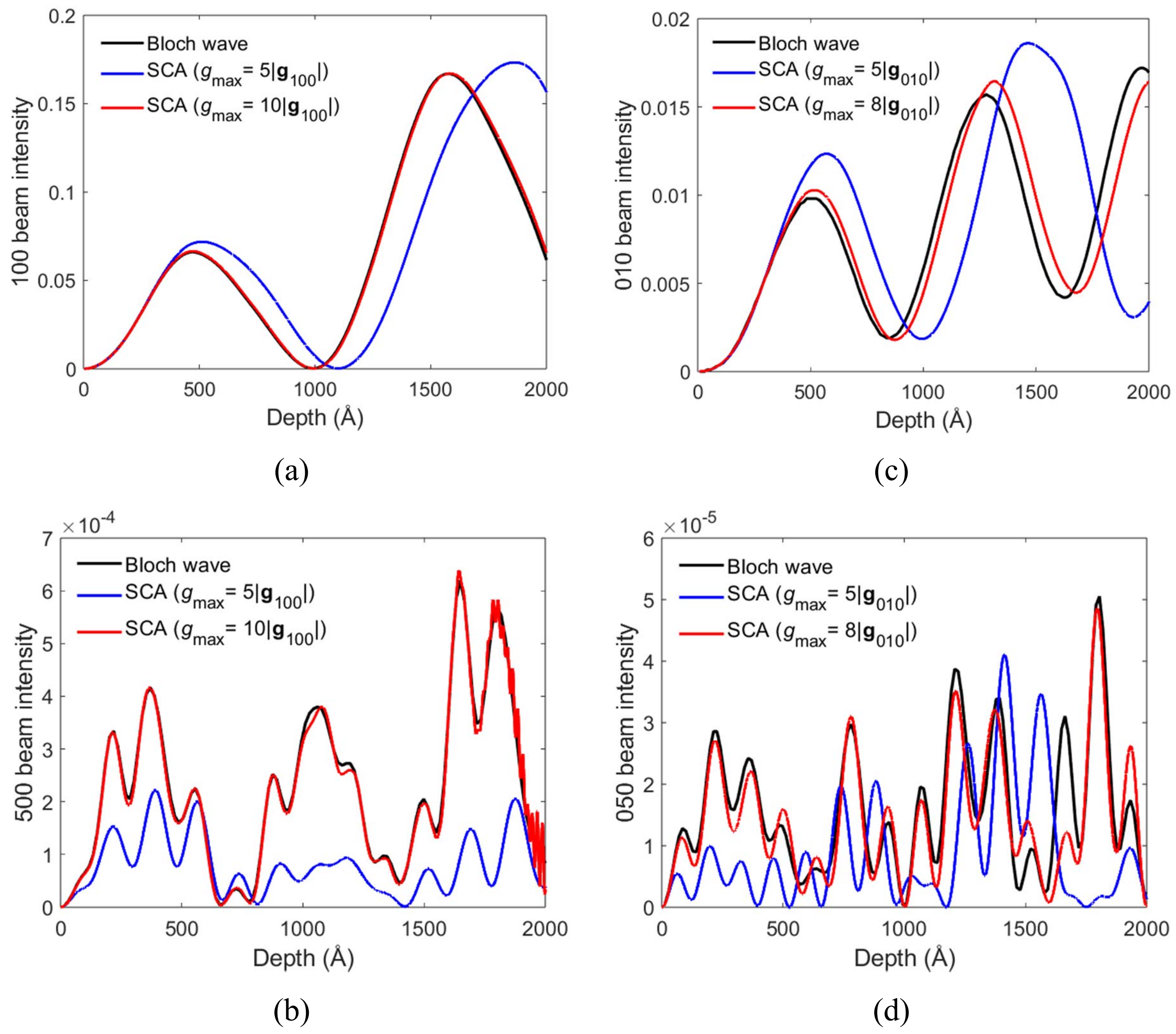

Figure S2: Comparison of normal beam incidence intensity pendellösung for [001]-TIPS pentacene and rubrene calculated using Bloch waves and scattering cluster algorithm (SCA). For the latter two different $g_{\max}$ values are shown. TIPS pentacene intensities are plotted for the (a) 100 and (b) 500 reflections, while for rubrene the intensities correspond to (c) 010, and (d) 050 beams.

(3) Effect of slice thickness Δ*z* on *R* factor

In Figure 4b (main text) the *R* factor in TIPS pentacene (normal beam incidence) was calculated as a function of specimen depth for a SCA slice thickness Δz of 0.2 Å and a SCA cluster size of $10|\mathbf{g}_{100}|$. The *R* factor increased rapidly for thicknesses larger than ~1000 Å. The overall convergence can however be improved by using smaller values of Δz. Figure S3 shows the corresponding *R* factor plot for Δz = 0.10 Å and Δz = 0.05 Å respectively, where the *R* factor is now smaller than 3% for all simulated specimen depths. The two Δz values also give approximately the same *R* factor for specimen thicknesses ≤1400 Å, indicating that the remaining discrepancy between SCA and Bloch waves is due to the finite size of the SCA cluster.

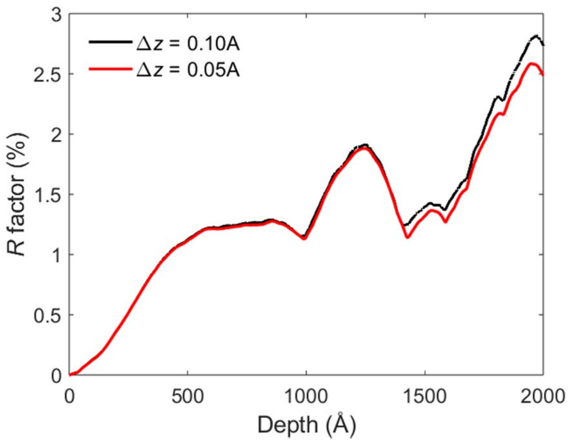

Figure S3: *R* factor plot for TIPS pentacene (normal beam incidence) as a function of specimen depth. The SCA cluster size is $10|\mathbf{g}_{100}|$. Plots for Δz values of 0.10 Å and 0.05 Å are shown.
